# One new species of *Pseudopoda* Jäger, 2000 from Shennongjia, Central China (Araneae, Sparassidae)

**DOI:** 10.3897/BDJ.12.e130445

**Published:** 2024-08-02

**Authors:** Lelei Wen, Changchun Li, Yang Zhong

**Affiliations:** 1 Hubei Key Laboratory of Quality Control of Characteristic Fruits and Vegetables, College of Life Science and Technology, Hubei Engineering University, Xiaogan 432000, China Hubei Key Laboratory of Quality Control of Characteristic Fruits and Vegetables, College of Life Science and Technology, Hubei Engineering University Xiaogan 432000 China; 2 School of Nuclear Technology and Chemistry & Biology, Hubei University of Science and Technology, Xianning 437100, China School of Nuclear Technology and Chemistry & Biology, Hubei University of Science and Technology Xianning 437100 China

**Keywords:** huntsman spiders, morphology, new species, taxonomy

## Abstract

**Background:**

*Pseudopoda* Jäger, 2000 is currently the largest genus in the family Sparassidae Bertkau, 1872, comprising 255 species. Of these, 154 species have been recorded in China, representing 60.4% of the global species.

In October 2023, a spider survey was conducted in Shennongjia National Nature Reserve, Hubei Province, China. After examination and morphological comparison, one new species of the genus *Pseudopoda* was identified and is described here.

**New information:**

In this paper, a new species of *Pseudopoda* collected in Hubei Province, China, is proposed and named *Pseudopodaguanmenshan* sp. nov. A detailed description, diagnosis, photographs and distribution map of the new species are provided.

## Introduction

The genus of *Pseudopoda* occurs in areas of South Asia (49 species in Nepal, India, Bhutan and Pakistan), East Asia (159 species in China and Japan) and Southeast Asia (53 species in Myanmar, Thailand, Laos and Vietnam) (WSC 2024). The goal of the present work is to describe a new species of *Pseudopoda* collected from Hubei Province, China.

To date, there are 154 species distributed in China and only four of these species have been described in Hubei Province (Quan et al. 2015; Gong et al. 2023; Zhang et al. 2023). In recent years, a series of taxonomic works on the genus have been reported (e.g. Deng et al. (2023); Gong et al. (2023); Zhang et al. (2023); Wu et al. (2024)). This genus can be characterised by the following characteristics: the conductor of the male palp is membranous (sometimes reduced or absent); the embolus is broadened and flattened, with the RTA arising proximally or mesially from the tibia; the epigyne has distinctly extending lateral lobes; and the internal duct system is covered by the first winding or the first winding and lateral lobes (modified from Jäger (2000); Jiang et al. (2018); Zhang et al. (2023)).

Shennongjia, located in the transition zone between the second and third terraces in China's Hubei Province, is endowed with extremely rich resources and is one of the most important ecological functional areas and ecological vulnerability zones in the world (Huang et al. 2023). It has attracted much attention for its superior natural ecological endowments (Cao et al. 2022). A survey of spiders in Shennongjia was carried out and resulted in the discovery of one new species, which is described herein.

## Materials and methods

The specimens were collected on leaves at night with bare hands. The type specimens are deposited in the School of Nuclear Technology and Chemistry & Biology, Hubei University of Science and Technology, Xianning, China. Specimens were preserved in 95% alcohol and examined using an Olympus SZX7 stereomicroscope. Left male palps were examined and photographed after dissection. Epigynes were removed and cleared in proteinase K at 56°C to dissolve non-chitinous tissues. The vulva was imaged after being embedded in Arabic gum. All photographs were captured with a KUY NICE industrial digital camera (20.0 megapixels), mounted on an Olympus CX43 dissecting microscope and assembled using Helicon Focus 3.10.3 image stacking software. All measurements were obtained using an Olympus SZX7 stereomicroscope and are given in millimetres. Eye diameters were measured at the widest part. The total body length does not include the chelicerae or spinnerets. Leg formula, spination and measurements of eye, palp and legs follow Jäger (2000) and Gong and Zhong (2020).

The abbreviations used in the text are: ALE = anterior lateral eye; AME = anterior median eye; AW = anterior width of carapace; BB = basal bulge of dorsal RTA; C = conductor; CO = copulatory opening; CH = clypeus height; dRTA = dorsal branch of RTA; E = embolus; EB = epigynal bulges; EP= embolic projection; F = finger-like tip of dorsal RTA; Fe = femur; FD = fertilisation duct; FW = first winding; LL = lateral lobes; Mt = metatarsus; MS = membranous sac; OL = opisthosoma length; OW = opisthosoma width; Pa = patella; PI = posterior incision of LL; PL = prosoma length; PLE = posterior lateral eyes; PME = posterior median eyes; PW = prosoma width; RTA = retrolateral tibial apophysis; S = spermatheca; Sp = spermophore; St = subtegulum; T = tegulum; Ti = tibia; I, II, III, IV = legs I to IV; vRTA = ventral branch of RTA.

## Taxon treatments

### Pseudopoda guanmenshan sp. nov.

3BDCEA1E-53EA-58DE-BBC1-1350EAF51AE3

103AC57F-B91A-4A77-A933-CD7F54B9807E

#### Materials

- **Type status:** Holotype. **Occurrence:** individualID: HUST-SPA-24-001; sex: male; **Taxon:** scientificName: *Pseudopodaguanmenshan* sp. nov.; **Location:** country: China; stateProvince: Hubei; locality: Muyu Town, Guanmenshan Scenic Area; verbatimElevation: 1200 m; verbatimLatitude: 31.45°N; verbatimLongitude: 110.40°E; **Identification:** identifiedBy: Yang Zhong; **Event:** samplingProtocol: by hand; year: 2023; month: October; day: 23
- **Type status:** Holotype. **Occurrence:** individualID: HUST-SPA-24-002-005; sex: 1 male, 3 females; **Taxon:** scientificName: *Pseudopodaguanmenshan* sp. nov.; **Location:** country: China; stateProvince: Hubei; locality: Muyu Town, Guanmenshan Scenic Area; verbatimElevation: 1200 m; verbatimLatitude: 31.45°N; verbatimLongitude: 110.40°E; **Identification:** identifiedBy: Yang Zhong; **Event:** samplingProtocol: by hand; year: 2023; month: October; day: 23

#### Description

**Figure 1.**
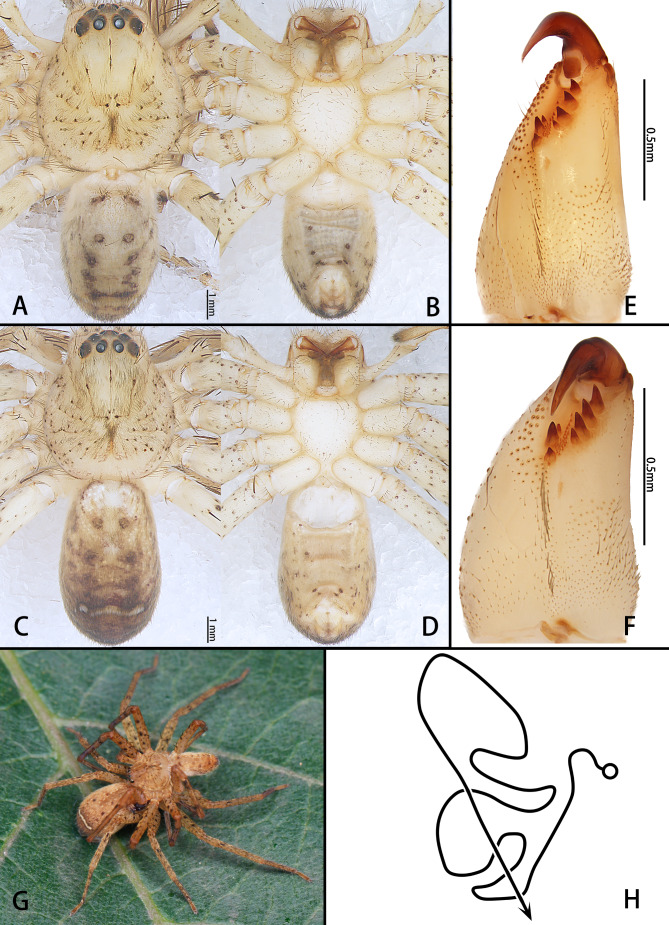
*Pseudopodaguanmenshan* sp. nov., habitus (A–D), cheliceral dentition (E, F), live specimens (G) and schematic course of internal duct system (H). A, B, E holotype male (HUST-SPA-24-001); C, D, F paratype female (HUST-SPA-24-002). A, C, G dorsal view; B, D, E , F ventral view. Scale bar: A–D 1 mm; E, F 0.5 mm.

**Male.** PL 5.0, PW 4.6, AW 2.7, OL 6.6, OW 4.7. Eyes: AME 0.24, ALE 0.34, PME 0.26, PLE 0.38, AME–AME 0.18, AME–ALE 0.07, PME–PME 0.24, PME–PLE 0.18, AME–PME 0.29, ALE–PLE 0.17, CH–AME 0.35, CH–ALE 0.47. Setation: Palp: 131, 101, 2101; Fe: I–III 323, IV 321; Pa: I–IV 101; Ti: I–II 2026, III–IV 2126; Mt: I–II 1014, III 2026, IV 3036. Measurements of palp and legs: Palp 6.4 (2.1, 0.7, 1.0, –, 2.6), I 19.3 (5.5, 1.3, 5.6, 5.1, 1.8), II 20.5 (5.9, 1.3, 6.1, 5.4, 1.8), III 15.4 (4.6, 1.1, 4.5, 3.7, 1.5), IV 16.9 (5.0, 1.0, 4.5, 4.6, 1.8). Leg formula: II-I-IV-III. The carapace is yellowish-brown dorsally, with black patches, shallow fovea and radial furrows. Chelicerae are pale reddish-brown. The sternum is yellow, with a few random black spots. Endites and labium are yellowish-brown. Legs are yellow, with brown dots randomly distributed and covered by short spines and setae. The dorsal opisthosoma is yellowish-brown with two pairs of dark muscle sigilla and a white transversal band in the posterior half. The ventral opisthosoma is uniformly yellowish-brown with some black patches (Fig. 1A and B). Chelicerae with three promarginal and four retromarginal teeth and with ~ 28 denticles (Fig. 1E).

**Figure 2.**
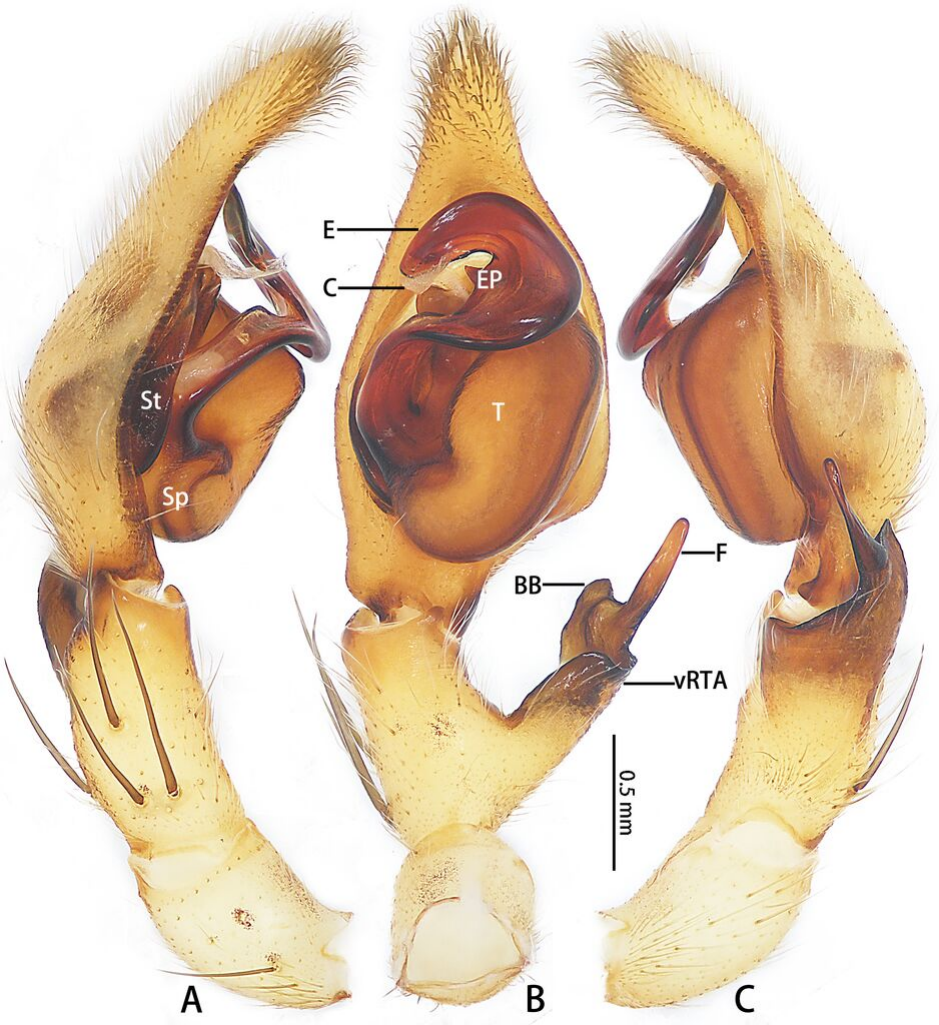
*Pseudopodaguanmenshan* sp. nov., male holotype (HUST-SPA-24-001), left palp (A–C), A prolateral view; B ventral view; C retrolateral view. Abbreviations: BB = basal bulge of dorsal RTA; C = conductor; E = embolus; EP = embolic projection; F = finger-like tip of dorsal RTA; Sp = spermophore; St = subtegulum; T = tegulum; vRTA = ventral branch of RTA. Scale bar: 0.5 mm.

Cymbium approximately 2.5 times the length of the tibia. Spermophore running submarginally and retrolaterally in tegulum. Conductor arising from tegulum at 12:00 o’clock position, covering the tip of embolus. Basal part of embolus broad, almost covering subtegulum, while the distal part tapers gradually and becomes trapezoidal at distal end. Embolic projection emerging at the middle margin of embolus as a pointed hump. Anterior margin of vRTA almost straight in ventral view (Fig. 2).

Female. PL 5.2, PW 4.5, AW 2.7, OL 5.4, OW 3.5. Eyes: AME 0.23, ALE 0.30, PME 0.26, PLE 0.32, AME–AME 0.20, AME–ALE 0.11, PME–PME 0.33, PME–PLE 0.26, AME–PME 0.25, ALE–PLE 0.26, CH AME 0.35, CH ALE 0.37. Setation: Palp: 131, 101, 2121, 1014; Fe: I–III 323, IV 321; Pa: I–IV 101; Ti: I–IV 2126; Mt: I–II 2024, III–IV 3036. Measurements of palp and legs: Palp 6.1 (2.0, 0.5, 1.3, –, 2.3), I 18.7 (5.4, 1.3, 5.4, 4.9, 1.7), II 19.4 (5.7, 1.3, 5.8, 5.0, 1.6), III 15.1 (4.8, 1.1, 4.3, 3.7, 1.2), IV 16.1 (5.0, 1.1, 4.1, 4.5, 1.4). Leg formula: II-I-IV-III. Chelicerae with three promarginal and four retromarginal teeth and with ~ 32 denticles (Fig. 1F).

**Figure 3.**
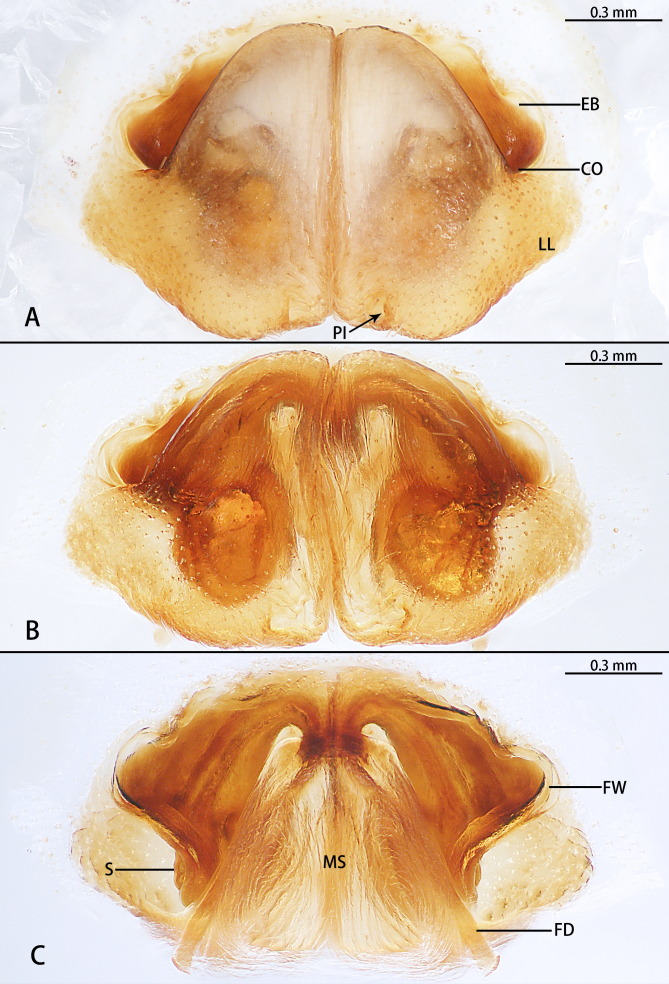
*Pseudopodaguanmenshan* sp. nov., female paratype (HUST-SPA-24-002), epigyne (A, B), vulva (C). A ventral view; B ventral view, macerated and embedded in Arabic gum; C dorsal view, macerated and embedded in Arabic gum. Abbreviations: CO = copulatory opening; EB = epigynal bulges; FD = fertilisation duct; FW = first winding; LL = lateral lobes; MS = membranous sac; PI = posterior incision of LL; S = spermatheca. Scale bar: 0.3 mm.

Epigynal field clearly wider than long; median margin of lateral lobes parallel and almost straight; posterior incision of lateral lobes and epigynal bulges distinct. Copulatory opening located at middle part of epigyne. In dorsal view, space between fertilisation ducts and first winding smaller than width of first winding, posterior end of first winding of internal duct system freely visible. Membranous sac between fertilisation ducts almost trapezoidal (Fig. 1H and Fig. 3).

Colouration as in males, opisthosoma brown dorsally (Fig. 1C and D).

#### Diagnosis

Males of *Pseudopodaguanmenshan* sp. nov. are similar to those of *P.tji* Jäger, 2015 (Jäger 2015: figs. 1−3) in having the dRTA with a long finger-like and bulged basal part, but it can be distinguished by the following combination of characters: (1) The embolus arising from tegulum at 8:00 o’clock position, with a triangular embolic projection (in *P.tji*, it is at the 9:00 o’clock position, without EP); (2) The tegulum is oval without tegular protrusion (the tegulum is oval, with TP in *P.tji*); and (3)The RTA arising basally from the tibia, with the vRTA lacking a rounded protrusion in retrolateral view (in *P.tji*, it is medially positioned with the vRTA having a rounded protrusion) (Fig. 2). The females of this species can be separated from other *Pseudopoda* species by their unique anterior margins of lateral lobes, which are almost ∩-shaped (Fig. 3A and B).

#### Etymology

The species name is derived from the name of the type locality; noun in apposition.

#### Distribution

**Figure 4.**
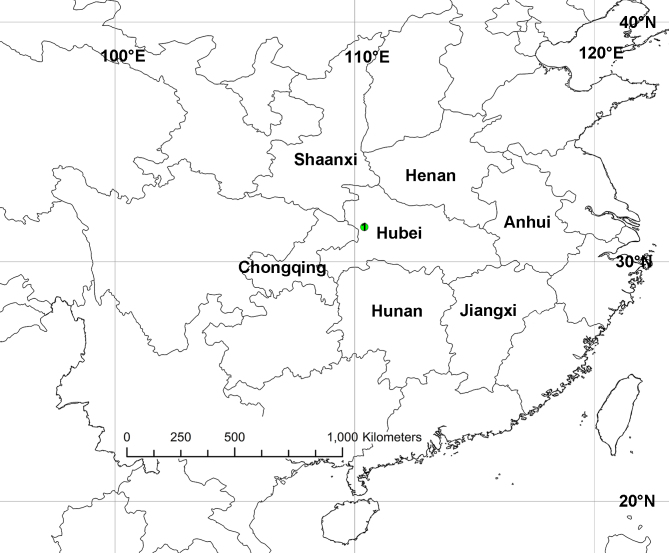
Collection locality of *Pseudopodaguanmenshan* sp. nov.

Known only from the type locality in Shennongjia, Hubei Province, China (Fig. 4).

#### Notes

We have presented the copulatory behaviour diagram for this newly-identified species within its native habitat (Fig. 1G).

## Supplementary Material

XML Treatment for Pseudopoda guanmenshan
